# Supplementary material for: DMRforPairs: identifying Differentially Methylated Regions between unique samples using array based methylation profiles
Source: BMC Bioinformatics. 2014 May 15;15:141. doi: 10.1186/1471-2105-15-141 (PMC4046028; doi:10.1186/1471-2105-15-141)
Supplement: Additional file 2 — DMRforPairs output for the comparison of A431-MCF7 and NA17018-NA17105. Please start from the HTML files in each folder. Available via the BMC Bioinformatics website. [file 1471-2105-15-141-S2.zip › 1394847754114233_MOESM2_ESM/A431_MCF7/figures/11542.pdf]

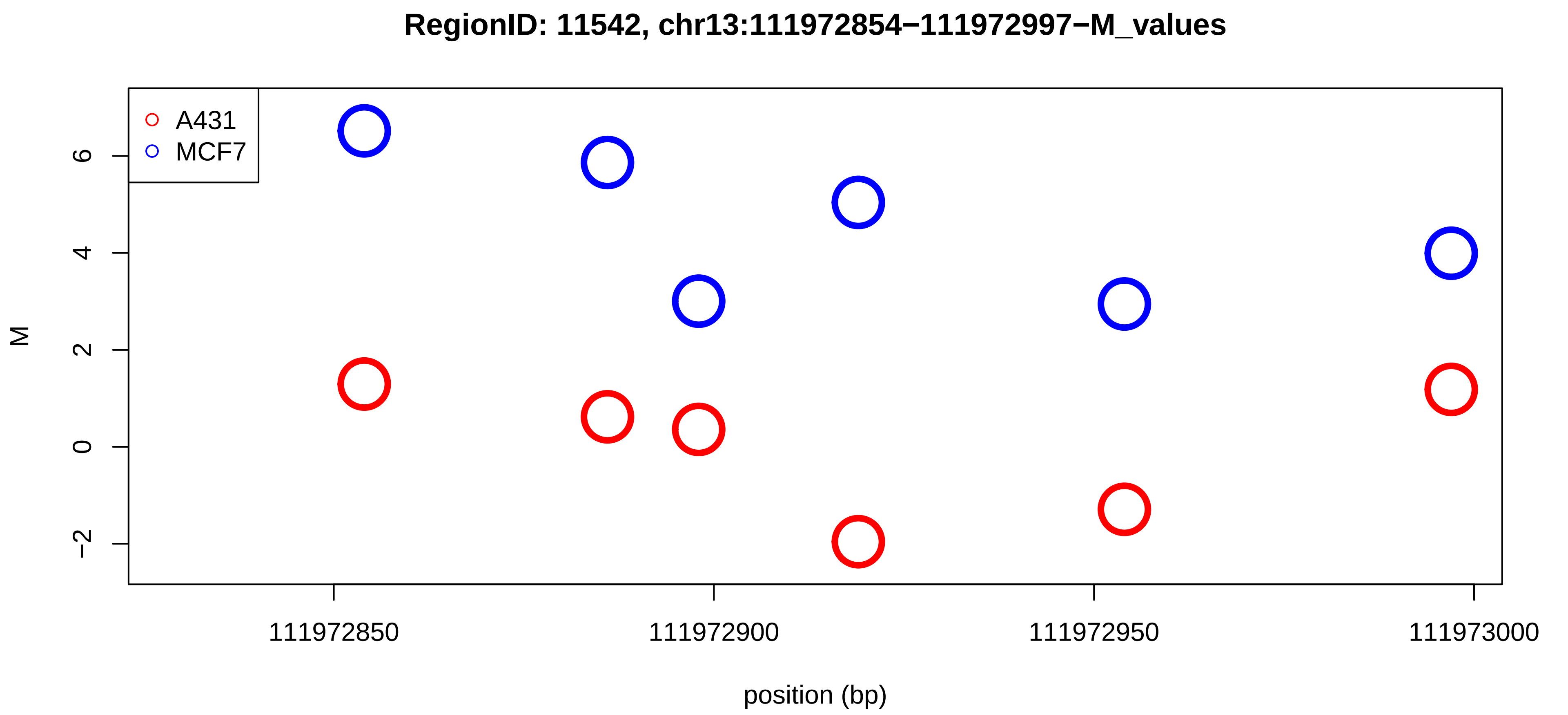
RegionID: 11542, chr13:111972854−111972997−M_values
A431
MCF7
M
−2
0
2
4
6
111972850
111972900
111972950
111973000
position (bp)

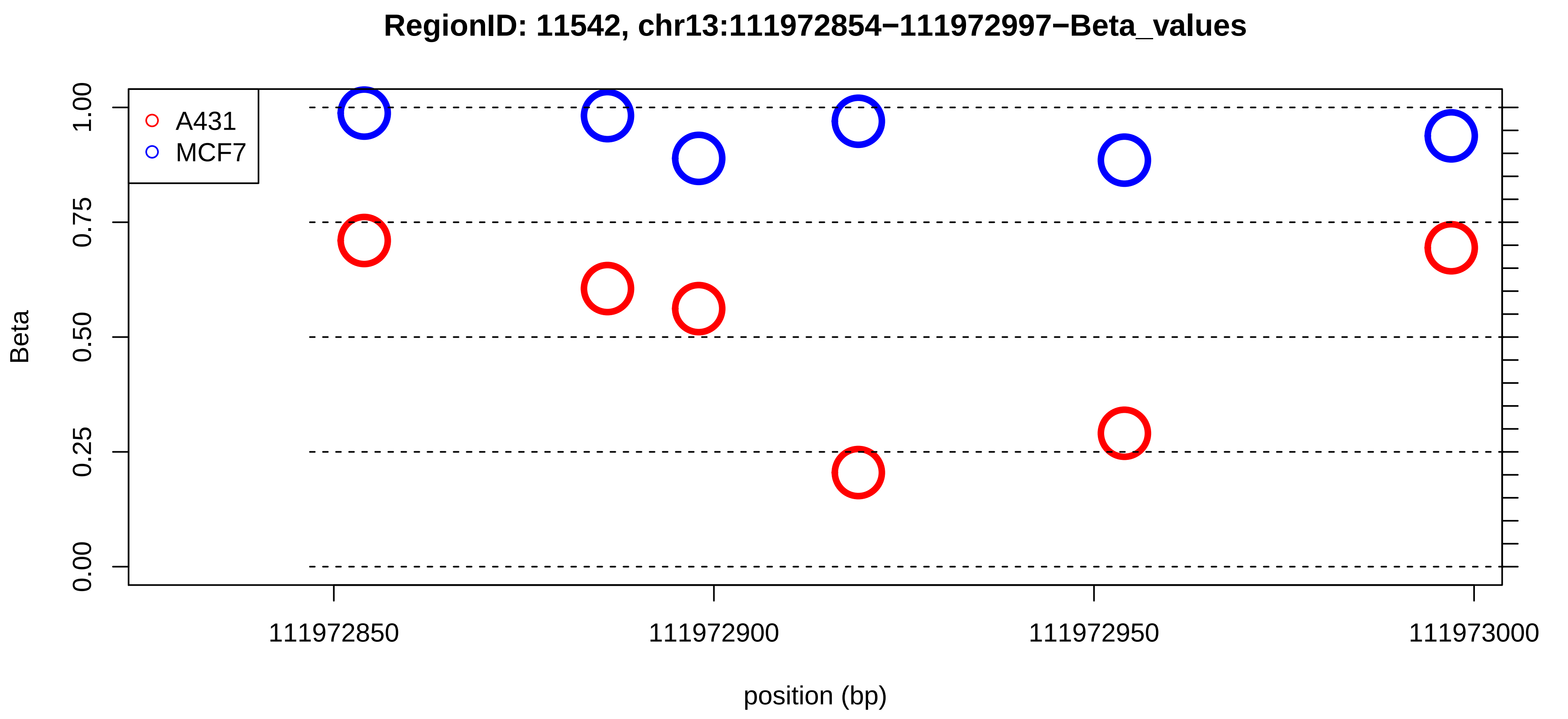
RegionID: 11542, chr13:111972854−111972997−Beta_values
A431
MCF7
Beta
0.00
0.25
0.50
0.75
1.00
111972850
111972900
111972950
111973000
position (bp)

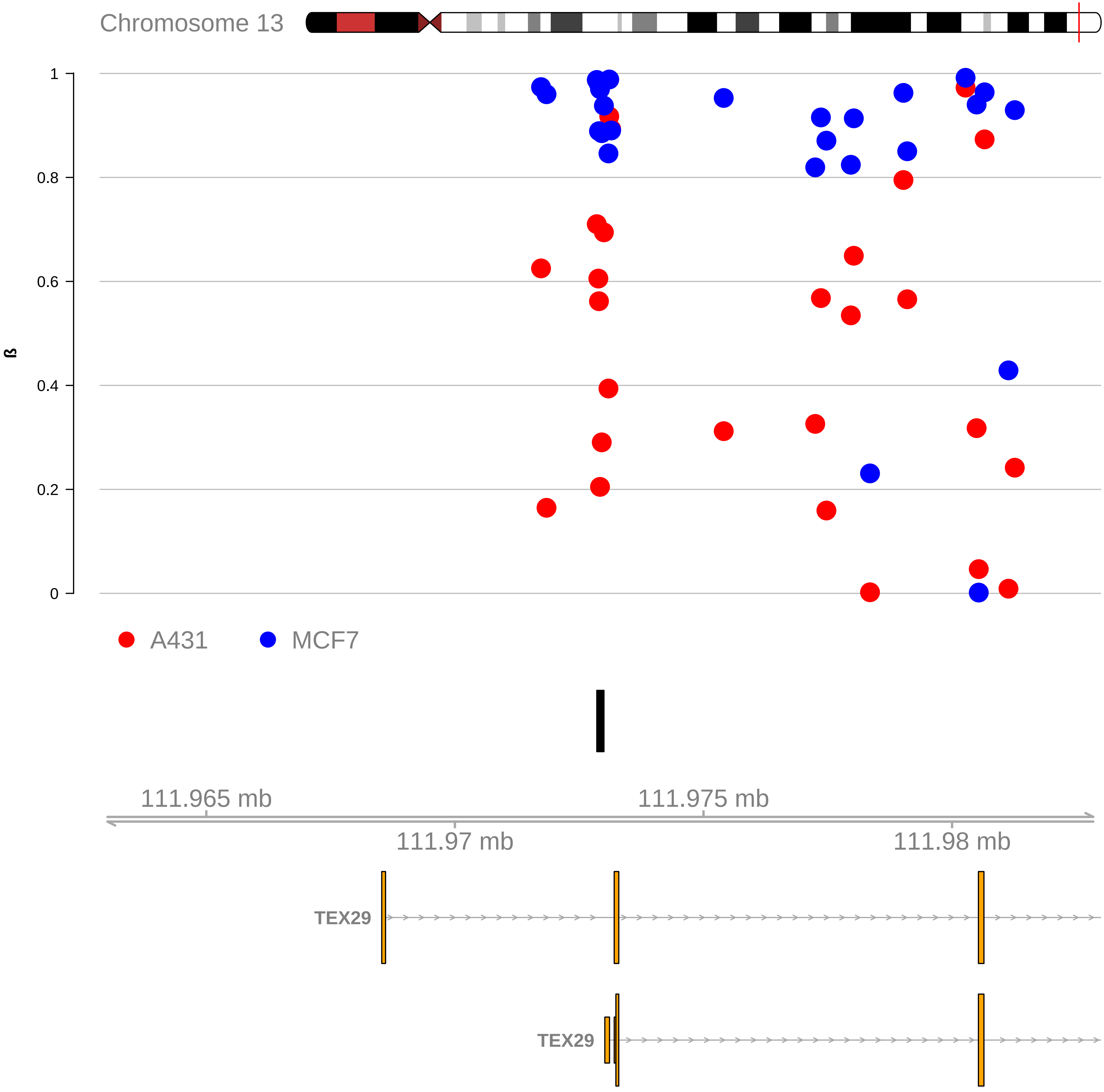
Chromosome 13
1
0.8
0.6
0.4
0.2
0
β
A431
MCF7
111.965 mb
111.975 mb
111.97 mb
111.98 mb
TEX29
TEX29
